# Agenesis of the Corpus Callosum and Skeletal Deformities in Two Unrelated Patients: Analysis via MRI and Radiography

**DOI:** 10.1155/2014/186973

**Published:** 2014-01-29

**Authors:** Ali Al Kaissi, Herbert Kurz, Wolfgang Bock, Gerald Pärtan, Klaus Klaushofer, Rudolf Ganger, Franz Grill

**Affiliations:** ^1^Ludwig Boltzmann Institute of Osteology at the Hanusch Hospital of WGKK and AUVA Trauma Centre Meidling, First Medical Department, Hanusch Hospital, Austria-Heinrich Collin Staße, 1140 Vienna, Austria; ^2^Orthopaedic Hospital of Speising, Paediatric Department, Speisinger Staße 109, 1130 Vienna, Austria; ^3^Department of Neonatology, Danube Hospital, Langobardenstraße 122, 1220 Vienna, Austria; ^4^Department of Pediatrics, Danube Hospital, Langobardenstraße 122, 1220 Vienna, Austria; ^5^Department of Radiology, Danube Hospital, Langobardenstraße 122, 1220 Vienna, Austria

## Abstract

*Purpose*. Mental retardation, mild to severe epilepsy and cerebral palsy often of hemiplegic type are common accompaniments in patients with agenesis/hypoplasia of the corpus callosum. Skeletal deformities of bilateral radiohumeral synostosis, brachydactyly, bilateral elbow dislocation, talipes equinovarus, and juxtacalcaneal accessory bones have been encountered in two unrelated children with agenesis of the corpus callosum. *Methods*. We report on two unrelated children who presented with the full clinical criteria of agenesis of the corpus callosum. Strikingly, both presented with variable upper and lower limb deformities. The clinical features, radiographic and MRI findings in our current patients, have been compared with previously reported cases identified through a PubMed literature review. *Results*. Bilateral radiohumeral synostosis associated with pyruvate dehydrogenase deficiency has been encountered in one patient. The other patient manifested bilateral elbow dislocation, coxa valga, talipes equinovarus, and bilateral juxtacalcaneal accessory bones. *Conclusion*. The constellation of malformation complexes in our current patients have the hitherto not been reported and expanding the spectrum of skeletal deformities in connection with agenesis of the corpus callosum.

## 1. Introduction

Partial or complete absence of the corpus callosum (CC) is among the commonest developmental anomalies of the brain. A large number of cerebral and other malformations occur in association with callosal agenesis. The brain anomalies include lipoma of the corpus callosum, cerebellar hypoplasia, the Dandy-Walker syndrome, hydrocephalus due to this or to obstruction of the foramen of Monro or aqueduct stenosis, interhemispheric cysts, porencephalic cysts, and the neuronal migration disorders [1, 2]. Associated craniofacial dysmorphic features include hypertelorism, encephaloceles, and craniosynostosis. Ocular anomalies seen include microphthalmia, coloboma, retinal lacunae (Aicardi syndrome), and optic nerve hypoplasia [3]. We describe two sporadic cases of unrelated girls with agenesis of the CC. The clinical features, radiographic and MRI findings in our current patients, have been compared with previously reported cases identified through a PubMed literature review.

## 2. Clinical Reports

### 2.1. Patient 1

A two-year-old girl was referred to our department because of bilateral and symmetrical contractures and shortenings of the upper limbs. She was born with low birth weight and she was hypotonic. Parents are not related and family history was noncontributory. At birth, she manifested severe psychomotor delay, hypotonia, seizures, and extensive asymmetrical cerebral atrophy and agenesis of the corpus callosum. Ophthalmological examination showed poor visual tracking, grossly disconjugate eye movements, poor pupillary responses, and blindness. A diagnosis of pyruvate dehydrogenase complex (PDH) deficiency was made based on marked diminished PDH activity in skin fibroblasts and muscle tissue. Specific immunoblot studies revealed normal E_1alpha_ and E_1beta_ subunits of the PDH complex, and molecular analysis of the E_1alpha_ cDNA failed to demonstrate any mutation. The parents had no family history of PDH deficiency or unexplained fetal or neonatal deaths.

**Figure 1:**
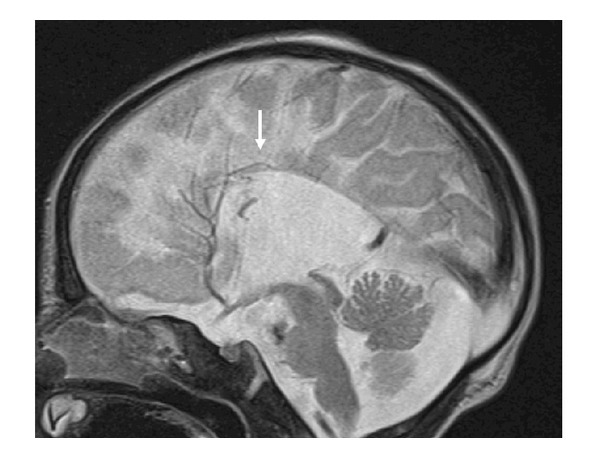
(Patient 1) Sagittal T2 TSE image shows agenesis of the corpus callosum (white arrow).

**Figure 2:**
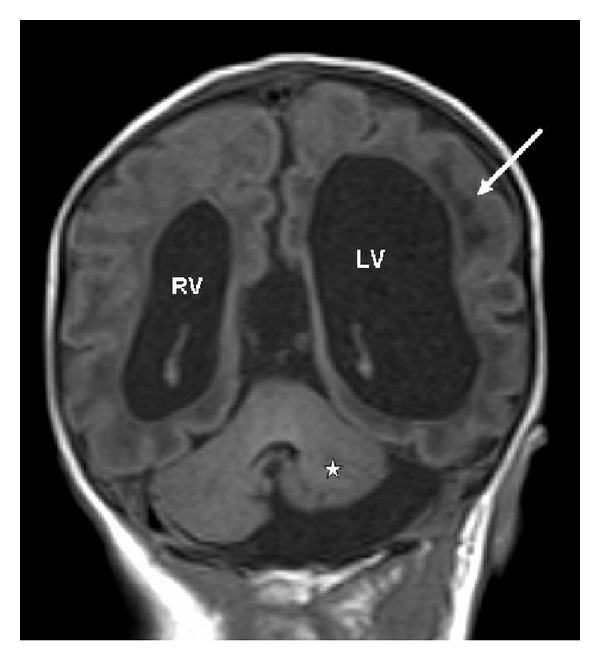
(Patient 1) Coronal T1 SE image shows subcortical leucencephalopathy (white arrow), marked enlargement of the left (LV) more than the right ventricle (RV), and hypoplasia predominantly of the left cerebellum (asterisk).

**Figure 3:**
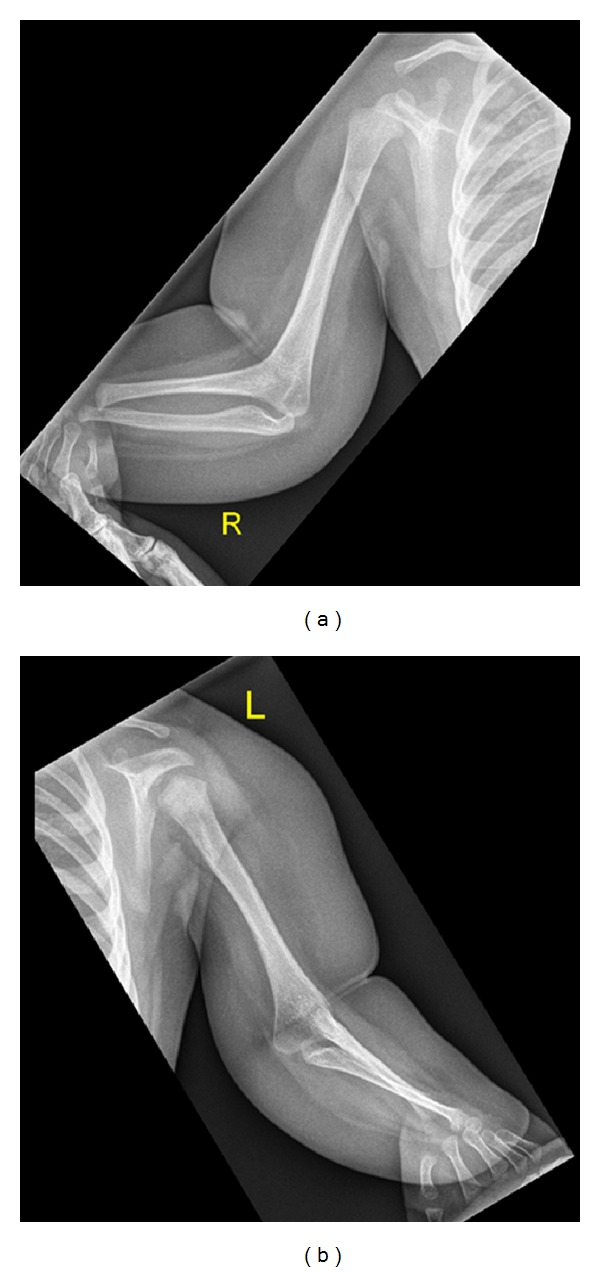
(Patient 1) Anteroposterior upper limb radiograph shows bilateral and symmetrical humeroradial synostosis associated with ulnar ray hypoplasia.

**Figure 4:**
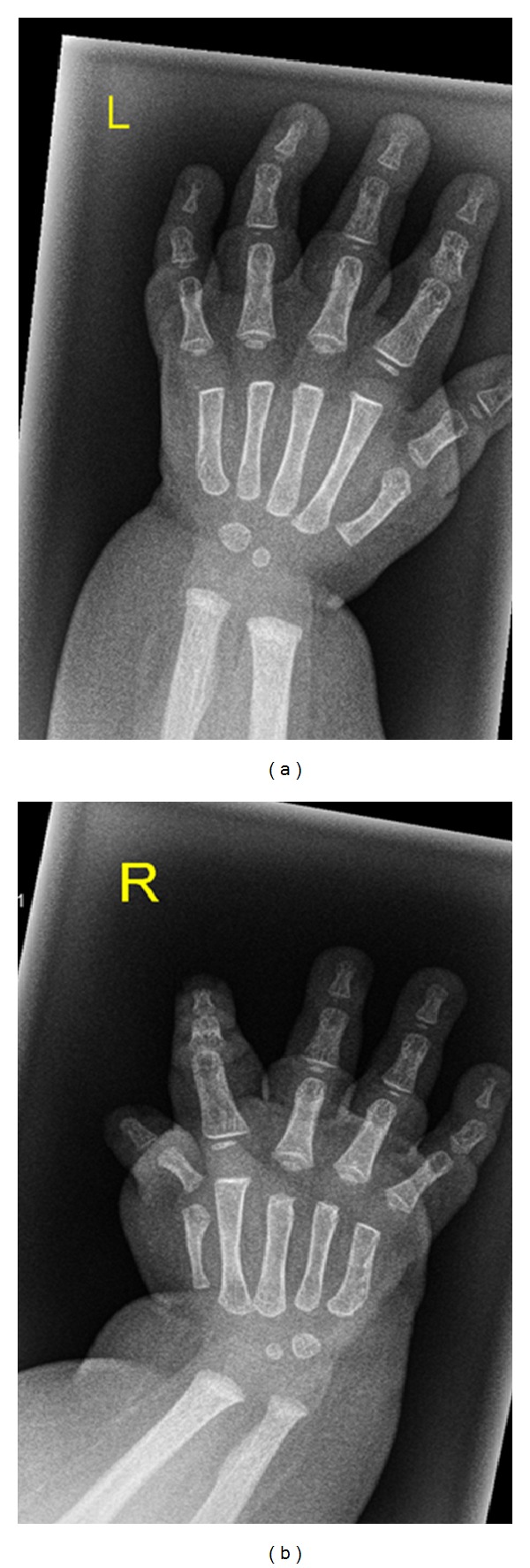
(Patient 1) Anteroposterior hand radiographs showed brachydactyly dysplastic first and second digits and clinodactyly of the 5th fingers.

Clinical examination showed growth around the 10th percentile; the head circumference was around the 50th percentile, rounded facies, frontal bossing, and upturned hypoplastic nose. MRI one day after birth showed agenesis of the corpus callosum with colpocephalic enlargement of the side ventricles (Figure 1). Coronal T1 SE image shows subcortical leucencephalopathy (white arrow) associated with marked enlargement of the left (LV) more than the right ventricle (RV), and hypoplasia predominantly of the left cerebellum (asterisk) (Figure 2). Anteroposterior upper limb radiograph shows bilateral and symmetrical humeroradial synostosis associated with ulnar ray hypoplasia (Figure 3). Anteroposterior hand radiographs showed dysplastic first and second digits, respectively (Figure 4).

### 2.2. Patient 2

**Figure 5:**
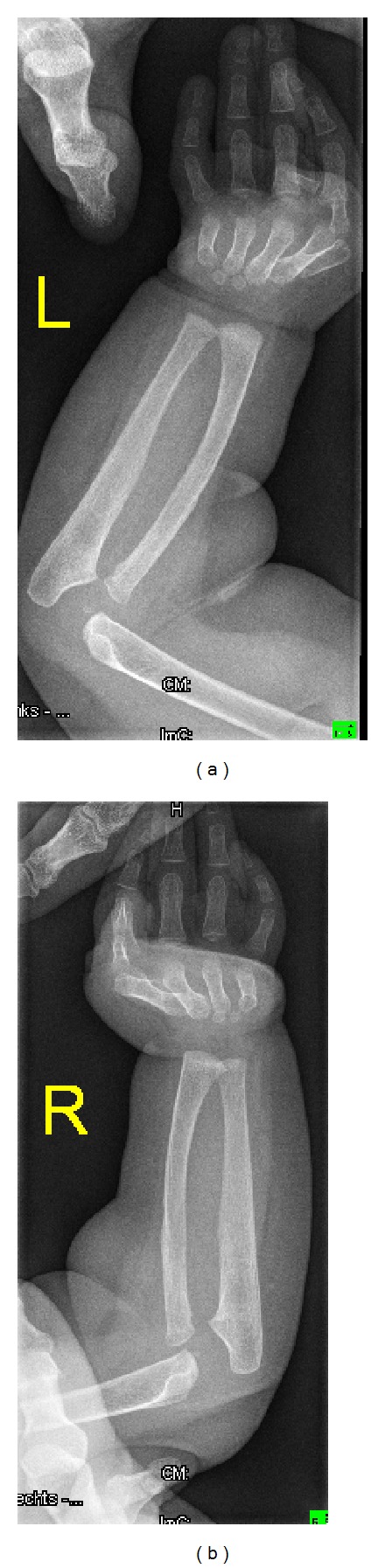
(Patient 2) AP forearms radiograph showed bilateral dislocation of the elbows, dysplastic ulnae, and brachydactyly.

**Figure 6:**
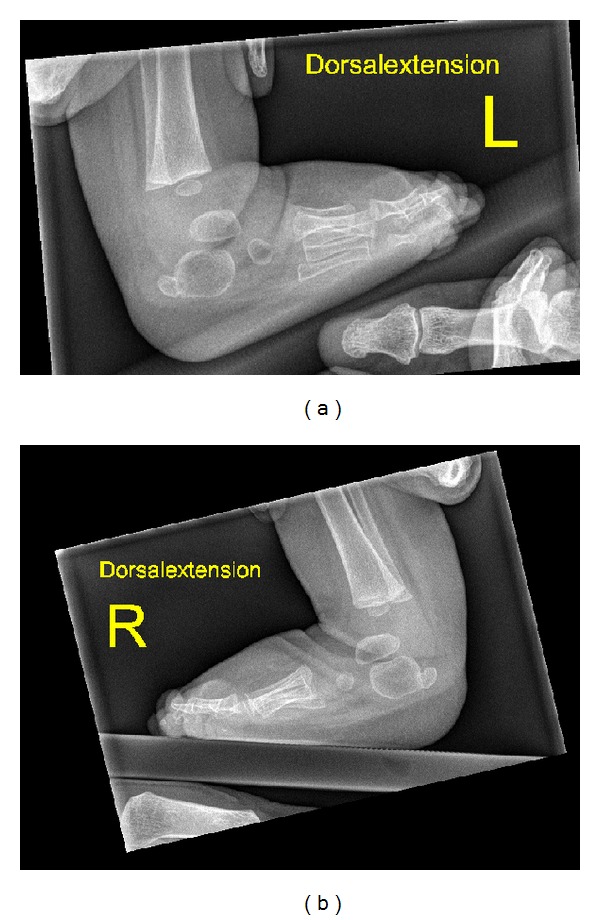
(Patient 2) Lateral feet radiograph showed the juxtacalcaneal ossific centres.

A two-year-old girl was referred to our department because of bilateral elbow dislocation and bilateral club foot. She was born with low birth weight and she was hypotonic. Parents are not related and family history was noncontributory. At birth, she manifested severe psychomotor delay, hypotonia, and seizures, and MRI scan showed hypoplasia of the corpus callosum. Ophthalmological examination showed normal vision. Investigations showed normal serum levels of luteinizing hormone, follicle stimulating hormone, prolactin, estradiol, and progesterone hormone. Karyotyping of the patient and their parents were normal. Metabolic parameters to assess blood glucose, uric acid, serum calcium, phosphorus, and parathyroid hormone levels were normal. She exhibited brachycephaly, rounded facies, hypertelorism, and micrognathia. Anteroposterior (AP) elbows radiograph showed bilateral elbow dislocation, hypoplasia of the ulnae, and brachydactyly (Figure 5). AP pelvis radiograph showed coxa valga. Lateral foot radiograph showed (talipes equinovarus) associated with a juxtacalcaneal accessory bone (bilaterally) (Figure 6).

## 3. Discussion 

The aetiology of agenesis/hypoplasia of the CC is usually unknown, but the defect has been noted with various chromosomal aberrations, in association with severe subnormality and usually early death [1, 2]. Various syndromic entities have been described in correlation with agenesis/hypoplasia of the CC. El Abd et al. [4] described a Bedouin family with Greig cephalopolysyndactyly syndrome. The index case and his father shared pre- and postaxial polysyndactyly associated with dysgenesis of the corpus callosum.

In acrocallosal syndrome, agenesis of CC is usually associated with combined immunodeficiency, foot anomalies (duplicated halluces), and postaxial polydactyly of the toes. Hand anomalies such as polydactyly and bifid terminal phalanges of the thumbs are characteristic features.

Agenesis/hypoplasia of the (CC) has been described in connection with several recognized syndromic entities such as FG syndrome, Gorlin syndrome, Joubert syndrome, Lennox Gastaut syndrome, Rubinstein-Taybi syndrome, Turner syndrome, Williams syndrome, neurofibromatosis type I, and von Voss syndrome [3, 5–8]. None of the above mentioned entities seems similar to our patients.

The pyruvate dehydrogenase complex (PDHc) is an intramitochondrial multienzyme system, which plays a key role in aerobic glucose metabolism by catalysing the oxidative decarboxylation of pyruvate to acetyl-CoA. Genetic defects in the PDHc lead to lactic acidemia and neurological abnormalities. In the majority of the cases, the defect appears to reside in the E_1alpha_ subunit, the first catalytic component of the complex.

PDH has a significant effect on fetal development and this may become apparent during late pregnancy with poor fetal weight gain and falling maternal urinary oestriol levels. Delivery may be complicated and the babies may have a low Apgar scores. Patients with PDH deficiency typically develop symptoms soon after birth. In general, there are two forms of presentation, metabolic and neurological, and these occur at approximately equal frequency. The metabolic form presents as severe lactic acidosis with blood lactate concentration often more than 10 mmol/L. In a very few cases, the lactic acidosis has been reported to respond to high doses of thiamine [9–15]. When the metabolic abnormalities are less severe, the onset may be delayed until later in infancy and the clinical course is often characterised by intermediate episodes of lactic acidosis, often precipitated by intercurrent illness, and associated with cerebellar ataxia. A number of the patients with primarily neurological symptoms often fit into the category of Leigh's syndrome, a subacute neurodegenerative disease with prominent brain stem features [16].

There have been some reports that described the extent of malformation complex in connection with PDH deficiency. A low birth weight is very common. It has been suggested that there is a characteristic dysmorphic appearance associated with PDH deficiency, with a narrow head, frontal bossing, wide nasal bridge, long philtrum, and flared nostrils, but this is not seen in all cases and is not specific to the disease. Other congenital malformations such as simian creases, short neck, slight shortening of the limbs, flexion contractures, pes cavus, talipes, ventricular septal defects, and hydronephrosis have been described in few cases [17].

Humeroradial synostoses have been associated with the following syndromes: SC-phocomelia, Robert's syndrome, Apert syndrome, Cornelia de Lange, and the Antley-Bixler syndrome [3]. Cases associated with these syndromes are unlikely to reach sexual maturity and therefore do not pass on the defect. Humeroradial synostosis with ulnar aplasia has been reported by several authors. The majority of cases are unilateral. When both upper limbs arms are involved, cases with oligodactyly often have asymmetrical limb deficiencies [14]. Limb deficiencies have been reported in several infants exposed prenatally to cocaine and have been inducible in animal models [18]. Other reports showed the heritable pattern of the humeroradial synostosis. Pfeiffer and Braun-Quentin [19] reported two infants with humeroradial synostosis and absent ulnae, associated with oligodactyly. They suggest that some cases of humeroradial synostosis without abnormalities of the hands, but sometimes with hypoplasia of the fibulae and patellae, may represent a distinct autosomal recessive syndrome. None of the above mentioned cases showed humeroradial synostosis in association with pyruvate dehydrogenase deficiency.

Bilateral elbow dislocation and juxtacalcaneal accessory bones are usual features in patients with Larsen syndrome [3]. Larsen's syndrome is a rare inherited defect of connective tissue that is transmitted in both an autosomal dominant and recessive pattern. Larsen syndrome is characterized by its cardinal findings, which consist of multiple congenital joint dislocations, usually of the hips, knees, and elbows. Facially, there is frontal bossing, a depressed nasal bridge, hypertelorism, and a flat facies. Deformities of the fingers (spatulate) and calcaneus (a double ossification centre), as well as spinal anomalies that may lead to major spinal instability and spinal cord injury, are important characteristics. The overall phenotypic features in this patient were not compatible with Larsen syndrome.

## 4. In Conclusion 

Distinctive features in our two patients include rounded facies, hypertelorism, frontal bossing, and upturned hypoplastic nose. Further noteworthy features in the first patient were bilateral and symmetrical humeroradial synostosis associated with ulnar ray hypoplasia and dysplastic digits. Antenatal brain damage is a well known malformation in patients with prenatal pyruvate dehydrogenase deficiency. The second child showed bilateral elbow dislocation, brachydactyly, and juxtacalcaneal accessory bones. Depending on the relevant literature search we present for the first time two unrelated patients with a unique constellation of malformation complex. But, nevertheless we have not been able to elucidate the actual pathogenesis between the variable skeletal deformities in these two patients and agenesis of the corpus callosum.
